# Diverticular disease and risk of incident major adverse cardiovascular events: a nationwide matched cohort study

**DOI:** 10.1093/ehjqcco/qcae074

**Published:** 2024-08-22

**Authors:** Anders Forss, Wenjie Ma, Marcus Thuresson, Jiangwei Sun, Fahim Ebrahimi, David Bergman, Ola Olén, Johan Sundström, Jonas F Ludvigsson

**Affiliations:** Department of Medical Epidemiology and Biostatistics, Karolinska Institutet, Nobels Väg 12 A, 171 77 Stockholm, Sweden; Gastroenterology Unit, Department of Gastroenterology, Dermatovenereology and Rheumatology, Karolinska University Hospital, 171 76 Stockholm, Sweden; Clinical and Translational Epidemiology Unit, Massachusetts General Hospital and Harvard Medical School, Boston, MA 02114, USA; Division of Gastroenterology, Massachusetts General Hospital and Harvard Medical School, Boston, MA 02114, USA; Department of Medical Epidemiology and Biostatistics, Karolinska Institutet, Nobels Väg 12 A, 171 77 Stockholm, Sweden; Statisticon, AB, 753 22 Uppsala, Sweden; Department of Medical Epidemiology and Biostatistics, Karolinska Institutet, Nobels Väg 12 A, 171 77 Stockholm, Sweden; Department of Medical Epidemiology and Biostatistics, Karolinska Institutet, Nobels Väg 12 A, 171 77 Stockholm, Sweden; Department of Gastroenterology and Hepatology, University Digestive Health Care Center Basel—Clarunis, 4002 Basel, Switzerland; Department of Medical Epidemiology and Biostatistics, Karolinska Institutet, Nobels Väg 12 A, 171 77 Stockholm, Sweden; Clinical Epidemiology Division, Department of Medicine Solna, Karolinska Institutet, 171 77 Stockholm, Sweden; Sachs’ Children and Youth Hospital, Stockholm South General Hospital, Stockholm, Sweden; Department of Clinical Science and Education Södersjukhuset, Karolinska Institutet, 118 61 Stockholm, Sweden; Department of Medical Sciences, Uppsala University, 752 37 Uppsala, Sweden; The George Institute for Global Health, University of New South Wales, Sydney, 2000 News South Wales, Australia; Department of Medical Epidemiology and Biostatistics, Karolinska Institutet, Nobels Väg 12 A, 171 77 Stockholm, Sweden; Department of Paediatrics, Örebro University Hospital, 703 83 Örebro, Sweden; Department of Medicine, Columbia University College of Physicians and Surgeons, New York, NY 10032, USA

**Keywords:** Biopsy, Diverticular disease, Epidemiology, Register-based, Cardiovascular

## Abstract

**Background:**

An increased risk of cardiovascular disease (CVD) has been reported in patients with diverticular disease (DD). However, there are knowledge gaps about specific risks of each major adverse cardiovascular event (MACE) component.

**Methods and results:**

This nationwide cohort study included Swedish adults with DD (1987–2017, *N* = 52 468) without previous CVD. DD was defined through ICD codes in the National Patient Register and colorectal histopathology reports from the ESPRESSO study. DD cases were matched by age, sex, calendar year, and county of residence to ≤5 population reference individuals (*N* = 194 525). Multivariable-adjusted hazard ratios (aHRs) for MACE up until December 2021 were calculated using stratified Cox proportional hazard models. Median age at DD diagnosis was 62 years, and 61% were females. During a median follow-up of 8.6 years, 16 147 incident MACE occurred in individuals with DD and 48 134 in reference individuals [incidence rates (IRs)= 61.4 vs. 43.8/1000 person-years], corresponding to an aHR of 1.24 (95%CI = 1.22–1.27), equivalent to one extra case of MACE for every 6 DD patients followed for 10 years. The risk was increased for ischaemic heart disease (IR = 27.9 vs. 18.6; aHR = 1.36, 95%CI = 1.32–1.40), congestive heart failure (IR = 23.2 vs. 15.8; aHR = 1.26, 95%CI = 1.22–1.31), and stroke (IR = 18.0 vs. 13.7; aHR = 1.15, 95%CI = 1.11–1.19). DD was not associated with cardiovascular mortality (IR = 18.9 vs. 15.3; aHR = 1.01, 95%CI = 0.98–1.05). Results remained robust in sibling-controlled analyses.

**Conclusions:**

Patients with DD had a 24% increased risk of MACE compared with reference individuals, but no increased cardiovascular mortality. Future research should confirm these data and examine underlying mechanisms and shared risk factors between DD and CVD.

## Graphical Abstract

**Graphical Abstract:**
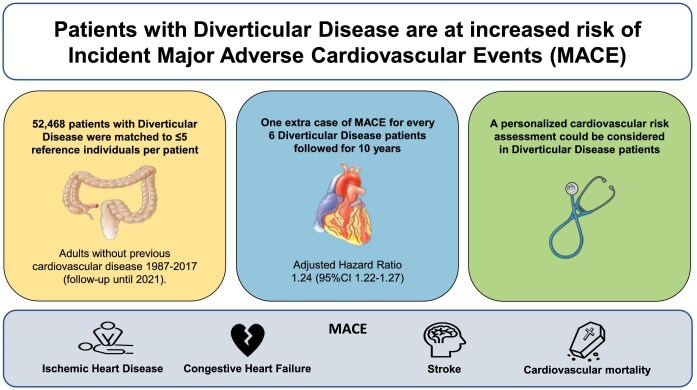

Key learning points **What is already known**

- Diverticular disease has been associated with an increased risk of cardiovascular events.
- However, there is a lack of data on specific cardiovascular outcomes.

**What this study adds**

- This study of individuals with diverticular disease found an increased risk of congestive heart failure, ischaemic heart disease, and stroke, but no increased cardiovascular mortality.
- Assessment of cardiovascular risk factors could be considered in individuals with diverticular disease.
- Future research should examine potentially shared risk factors between diverticular disease and cardiovascular disease.

## Introduction

Colonic diverticular disease (DD) includes colonic diverticulae that are either non-inflamed (diverticulosis) or inflamed (diverticulitis). DD is common in Western countries, with almost one in five affected by the disease.^1^ In ages above 60 years, up to one-third is estimated to have diverticulosis.^2^ Approximately 4% of those with diverticulosis develop diverticulitis.^1,3^ Diverticulosis can be asymptomatic, while acutely inflamed diverticulae can cause emergencies such as perforation, infectious complications including abscesses and peritonitis, as well as lower gastrointestinal (GI) bleeding.^1,4^ The pathogenesis of DD is not fully understood but is believed to be linked to genetic predisposition, neuromuscular abnormalities, chronic low-grade inflammation or acute inflammation, and an altered colonic motility.^5^ Risk factors for DD include obesity, low-fibre diet, hypertension, smoking, and possibly alcohol consumption.^4^

Systemic inflammation has been linked to coronary artery disease as a driver of the atherosclerotic process.^6,7^ This association has been suggested also for GI diseases^8,9^ where some studies have reported a higher risk of cardiovascular disease (CVD) in individuals with DD.^10,11^ We hypothesized that DD,^12^ through chronic low-grade inflammation, increases the risk of CVD. To the best of our knowledge, no study has yet assessed the specific risks of individual components of major adverse cardiovascular events (MACE), comprising ischaemic heart disease (IHD), congestive heart failure (CHF), stroke, and cardiovascular mortality, within the same large population-based cohort.

Therefore, in this study, we aimed to investigate the risk of incident MACE and its components in a large nationwide cohort of individuals with DD in Sweden from 1987 to 2017.

## Methods

### Study population and case definitions

In this nationwide population-based matched cohort study, we used prospectively recorded histopathology data in the ESPRESSO (Epidemiology Strengthened by Histopathology Reports in Sweden) study^13^ to identify individuals with a recorded topographical (anatomical site) Systematised Nomenclature of Medicine (SnoMed) code for a colorectal biopsy report. The ESPRESSO study includes colorectal histopathology biopsy and surgical specimen reports submitted to all (*n* = 28) pathology departments in Sweden (1965–2017). It has a complete coverage of reports containing data on date and location, a description of topography (anatomical site) within the GI tract, and morphology (referring to changes in cells and tissues) according to the SnoMed system.

We included all individuals with hospital or outpatient clinic records identifying colonic diverticulae with International Classification of Disease (ICD) codes indicating DD (ICD-8: 562,10-562,11; 562,18-562,19; ICD-9: 562B; ICD-10: K57.2-K57.5, K57.8-K57.9) in the Swedish National Patient Register (NPR)^14^ and SnoMed topography codes (T codes) of the colorectum (T67-T68). The second of either the first ICD code for DD or colorectal histology report (both conditions had to be fulfilled) was considered the date of DD diagnosis (index date). Individuals with DD were categorized according to morphology codes (M codes) into those with diverticula form (M327, M32700, M32710, M46400, M4642) and acute, chronic, or unspecified inflammation in the colonic mucosa on histology (M40-44, M4000, M4100, M4211, M4300, M4502, M4500), and those with normal histology (M00100, M00110).

Through the unique personal identity number^15^ assigned to all Swedish legal residents, we linked ESPRESSO data with data on demographics, migration, death, education level, incident MACE, and comorbidities. We ascertained covariates and outcome measures from the NPR. The NPR includes prospectively recorded inpatient data (discharge diagnosis and procedure codes) since 1964, with a full nationwide coverage from 1987, and since 2001 also includes outpatient visits (except primary care data). Clinical disease diagnoses in the NPR exhibit positive predictive values between 85 and 95%, including CVD diagnoses.^14^

We matched each DD case by age, sex, calendar year, and county of residence with up to five general population reference individuals without DD or CVD identified from the Swedish Total Population Register (TPR).^16^ We excluded anyone with any previous diagnosis for CVD, inflammatory bowel disease, a procedure code of colectomy,^17^ or emigration, recorded before or on the index date (Supplementary material online, *Table S1*). Identical exclusion criteria were applied to both individuals with DD, reference individuals, and siblings. Individuals with DD and reference individuals were identified during 1987–2017 and followed to 31 December 2021. A flowchart of inclusion and exclusion is presented in Supplementary material online, *Figure S1*.

### Outcomes and covariates

The primary outcome was incident MACE, defined as a composite outcome of ≥1 primary or secondary inpatient or outpatient non-primary specialized care diagnosis (as registered in the NPR after the index date) of IHD, CHF, stroke, or cardiovascular mortality (Supplementary material online, *Table S2*). Secondary outcomes included each of the individual MACE components. Data in the Cause of Death Register (CDR)^18^ was used to ascertain cardiovascular mortality. In cases where one patient was diagnosed with more than one of the individual MACE components, this patient contributed to each outcome using the date of the respective diagnosis.

Demographic data, including age, sex, date of birth, county of residence at the index date, country of birth (two categories: Nordic, including Sweden, Denmark, Finland, Norway, and Iceland vs. other countries), and emigration status were obtained from the TPR,^16^ and education level was ascertained from the longitudinal integrated database for health insurance and labour market studies.^19^ Information about comorbidities was extracted from the NPR using ICD codes (Supplementary material online, *Table S3*),^14^ and the Multigeneration Register (part of the TPR)^20^ was used to gather information on siblings for full-sibling analysis.

### Statistics

Follow-up started at the index date and continued to the first diagnosis of either any incident MACE outcome, death, emigration, or end of the follow-up (31 December 2021). Incidence rates (IRs) and absolute rate differences (RD) per 1000 person-years (py) with 95% confidence intervals (95%CIs) were calculated for MACE and its components in patients with DD compared to reference individuals. We estimated multivariable-adjusted hazard ratios (aHRs) and 95%CIs for incident MACE using stratified Cox proportional hazards regression models. We treated competing events as censoring events. In multivariable statistical model we conditioned on the matching factors, along with *a priori* list of potential confounding risk factors for both DD and MACE, including chronic obstructive pulmonary disease (COPD) diagnosis in those aged ≥40 years as a proxy for heavy smoking, alcohol related diseases to account for a history of substantial alcohol use,^21^ as well as relevant comorbidities (obesity, hypertension, dyslipidaemia, diabetes, and chronic kidney disease) (for ICD codes see Supplementary material online, *Table S3*), educational level, and healthcare utilization 6–24 months before DD diagnosis (in four categories: 0, 1, 2–3, or ≥4 visits).

To account for potential confounding linked to shared genetics and early life environmental exposures, we performed full-sibling analysis. We compared DD patients with ≥1 full-sibling without a record of DD or CVD before the start of the follow-up. We conditioned on family and adjusted for covariates in the multivariable model but also for sex, age, and calendar year.

The associations between DD and MACE were investigated in stratified analyses according to sex, age, duration of follow-up, calendar period of the start of follow-up, country of birth, and any metabolic disease (diabetes, obesity, hypertension, and dyslipidaemia). We also performed sensitivity analyses in which we restricted the cohort to cases with DD as (1) main diagnosis or (2) diagnosis from an inpatient setting.

The proportional hazards assumption was assessed by examining Schoenfeld residuals related to time. Two-sided *P*-values of <0.05 were considered statistically significant. Statistical analyses were performed using R software (version 4.3.1, R Foundation for Statistical Computing, Vienna, Austria; survival package version 3.5–5 [Therneau, 2023, https://CRAN.R-project.org/package=survival]).

### Ethics

This study and the ESPRESSO cohort were approved by the Regional Ethics Committee, Stockholm, Sweden. This study was register-based, informed consent was therefore waived.

## Results

We identified 52 468 patients with DD and 194 525 matched general population reference individuals during 1987–2017. The median age at DD diagnosis was 62 years [interquartile range (IQR) 52–70], and the majority of participants were females (61%). Metabolic comorbidities (diabetes, obesity, hypertension, and dyslipidaemia) were more frequent in patients with DD than in reference individuals (20.2% vs. 8.9%). Additional cohort characteristics at the start of follow-up are presented in *Table 1*.

**Table 1:** Cohort characteristics in patients with diverticular disease and general population reference individuals at the start of follow-up (1987–2017)

	Reference individuals *N* = 194 525	Diverticular disease *N* = 52 468
Sex, *n* (%)		
Male	74 247 (38)	20 438 (39)
Female	120 278 (62)	32 030 (61)
Age, years		
Mean (SD)	59 (13)	61 (13)
Median (IQR)	60 (50–68)	62 (52–70)
Range, min-max	18–98	18–98
Age groups, years, *n* (%)		
<30	3 378 (2)	736 (1)
30–39	11 793 (6)	2 571 (5)
40–49	30 470 (16)	6 867 (13)
50–59	51 253 (26)	12 452 (24)
60–69	56 817 (29)	15 507 (30)
70–79	32 285 (17)	10 706 (20)
≥ 80	8 529 (4)	3 629 (7)
Country of birth, *n* (%)		
Nordic	176 944 (91)	49 557 (94)
Other	17 575 (9)	2 910 (6)
NA	6 (0)	1 (0)
Level of education, *n* (%)		
Compulsory school (≤9 years)	56 509 (29)	16 136 (31)
Upper secondary school (10–12 years)	74 783 (38)	20 782 (40)
College or university (≥13 years)	48 241 (25)	11 246 (21)
NA	14 992 (8)	4 304 (8)
Calendar year start of follow-up		
1987–1999	41 976 (22)	10 268 (20)
2000–2009	77 584 (40)	20 733 (40)
2010–2017	74 965 (39)	21 467 (41)
Time between first diagnosis and biopsy, years		
Median (IQR)		0.4 (0.0–4.7)
Number of healthcare visits^a^, *n* (%)		
0	123 850 (64)	19 594 (37)
1	27 999 (14)	9 445 (18)
2–3	22 908 (12)	10 187 (19)
≥4	19 768 (10)	13 242 (25)
Follow-up time, years		
Mean (SD)	11.0 (6.5)	9.7 (6.3)
Median (IQR)	9.8 (6.2–14.9)	8.6 (5.3–13.2)
Range, min-max	0.0–34.6	0.0–34.5
Follow-up time, *n* (%)		
<2 years	9 739 (5)	4689 (9)
2 to <10 years	90 043 (46)	26 344 (50)
≥10 years	94 743 (49)	21 435 (41)
Comorbidity at start of follow-up, *n* (%)		
≥1 metabolic disease^b^	17 310 (8.9)	10 581 (20.2)
Diabetes	6 083 (3.1)	2 818 (5.4)
Obesity	1744 (0.9)	1120 (2.1)
Hypertension	11 845 (6.1)	8331 (15.9)
Dyslipidaemia	2202 (1.1)	1378 (2.6)
Chronic kidney disease	2941 (1.5)	1883 (3.6)
COPD (diagnosis at age ≥40 years)	2038 (1.0)	1582 (3.0)
Alcohol related disease	4086 (2.1)	1739 (3.3)

COPD, chronic obstructive pulmonary disease; IQR, interquartile range; NA, data not available; SD, standard deviation.

a Between 6 and 24 months before start of follow-up.

b Includes ≥1 of diabetes, obesity, hypertension, and dyslipidaemia.

### Incident major adverse cardiovascular events

**Figure 1:**
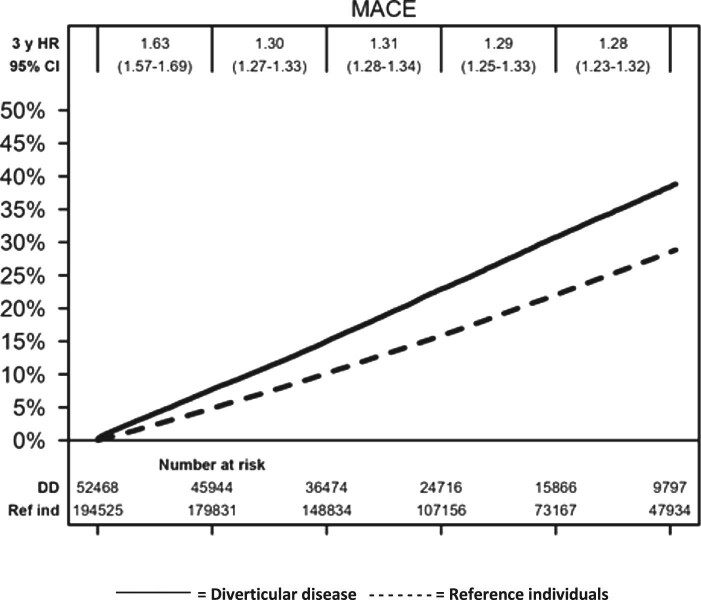
Kaplan-Meier curve (unadjusted) of the cumulative incidence of major adverse cardiovascular events (MACE) comprising ischaemic heart disease, congestive heart failure, stroke, and cardiovascular mortality) and hazard ratios (unadjusted) for patients with diverticular disease compared with population reference individuals per 3-year periods during 15 years of follow-up (1987 onwards). Y-axis shows the cumulative event risk and x-axis number of follow-up years (0–15 years). CI, confidence interval; HR, hazard ratio.

During a median follow-up of 8.6 years (IQR 5.3–13.2), we recorded 16 147 cases of incident MACE in patients with DD (IR = 61.4/1000 py) and 48 134 in reference individuals (IR = 43.8/1000 py), corresponding to an absolute RD of 17.5/1000 py) (*Table 2*, *Figure 1*), and equivalent to one extra case of MACE for every 6 individuals with DD followed for 10 years. After multivariable adjustment, patients with DD had a 24% higher relative risk of any MACE (aHR = 1.24, 95%CI = 1.22–1.27) compared with reference individuals. After additional adjustment for healthcare utilization, the risk increase was 20% (aHR = 1.20, 95%CI = 1.17–1.23) (*Table 2*).

**Table 2:** Incidence rates and hazard ratios for incident major adverse cardiovascular events in individuals with diverticular disease compared with general population reference individuals (1987–2021)

	Reference individuals *N* = 194 525	Diverticular disease *N* = 52 468
MACE^a^		
Incident events, n (%)	48 134 (24.7)	16 147 (30.8)
Incidence rate/1000 py (95% CI)	43.8 (43.5–44.2)	61.4 (60.4–62.3)
Absolute rate difference/1000 py (95% CI)	0 (ref)	17.5 (17.0–18.1)
Unadjusted HR	1 (ref)	1.45 (1.42–1.48)
Stratified HR^b^	1 (ref)	1.33 (1.30–1.36)
Adjusted HR^c^	1 (ref)	1.24 (1.22–1.27)
Adjusted HR^d^ including healthcare visits	1 (ref)	1.20 (1.17–1.23)

CI, confidence interval; HR, hazard ratio; MACE, major adverse cardiovascular events; py, person-years.

a Includes ischaemic heart disease, congestive heart failure, stroke, and cardiovascular mortality.

b Stratified on matched pairs.

c Adjusted for age, sex, calendar year, county of residence, country of birth (Nordic country or other), educational level (compulsory school, upper secondary school, or college/university), ≥1 metabolic disease (diabetes, obesity, hypertension, and dyslipidaemia), chronic kidney disease, chronic obstructive pulmonary disease with diagnosis ≥40 years of age, and alcohol related disease.

d Additionally adjusted for number of healthcare visits between 6 and 24 months before start of follow-up.

### Individual incident major adverse cardiovascular events

Individuals with DD showed elevated rates and relative risks for IHD (RD = 9.3/1000 py; aHR = 1.36, 95%CI = 1.32–1.40), CHF (RD = 7.5/1000 py; aHR = 1.26, 95%CI = 1.22–1.31), and stroke (RD = 4.3/1000 py; aHR = 1.15, 95%CI = 1.11–1.19), but not for cardiovascular mortality (RD = 3.6/1000 py; aHR = 1.01, 95%CI = 0.98–1.05) compared with reference individuals. After additional adjustment for healthcare utilization 6 to 24 months before baseline, estimates remained largely unchanged (*Table 3* and Supplementary material online, *Figure S2*A–D).

**Table 3:** Incidence rates and hazard ratios for incident ischaemic heart disease, cardiac heart failure, stroke, and cardiovascular mortality in individuals with diverticular disease compared with general population reference individuals (1987–2021)

	Reference individuals *N* = 194 525	Diverticular disease *N* = 52 468
Ischaemic heart disease		
Incident events, *n* (%)	20 998 (10.8)	7619 (14.5)
Incidence rate/1000 py (95% CI)	18.6 (18.4–18.9)	27.9 (27.3–28.5)
Absolute rate difference/1000 py (95% CI)	0 (ref)	9.3 (8.9–9.6)
Unadjusted HR	1 (ref)	1.53 (1.49–1.57)
Stratified HR^a^	1 (ref)	1.44 (1.40–1.48)
Adjusted HR^b^	1 (ref)	1.36 (1.32–1.40)
Adjusted HR including healthcare visits^c^	1 (ref)	1.31 (1.27–1.35)
Congestive heart failure		
Incident events, *n* (%)	18 259 (9.4)	6 590 (12.6)
Incidence rate/1000 py (95% CI)	15.8 (15.5–16.0)	23.2 (22.7–23.8)
Absolute rate difference/1000 py (95% CI)	0 (ref)	7.5 (7.1–7.8)
Unadjusted HR	1 (ref)	1.54 (1.49–1.58)
Stratified HR^a^	1 (ref)	1.37 (1.33–1.42)
Adjusted HR^b^	1 (ref)	1.26 (1.22–1.31)
Adjusted HR including healthcare visits^c^	1 (ref)	1.22 (1.18–1.26)
Stroke		
Incident events, *n* (%)	15 820 (8.1)	5 081 (9.7)
Incidence rate/1000 py (95% CI)	13.7 (13.5–13.9)	18.0 (17.5–18.5)
Absolute rate difference/1000 py (95% CI)	0 (ref)	4.3 (4.0–4.6)
Unadjusted HR	1 (ref)	1.34 (1.30–1.39)
Stratified HR^a^	1 (ref)	1.22 (1.17–1.26)
Adjusted HR^b^	1 (ref)	1.15 (1.11–1.19)
Adjusted HR including healthcare visits^c^	1 (ref)	1.12 (1.08–1.16)
Cardiovascular mortality		
Incident events, *n* (%)	18 227 (9.4)	5 568 (10.6)
Incidence rate/1000 py (95% CI)	15.3 (15.1–15.6)	18.9 (18.4–19.4)
Absolute rate difference/1000 py (95% CI)	0 (ref)	3.6 (3.3–3.9)
Unadjusted HR	1 (ref)	1.29 (1.25–1.33)
Stratified HR^a^	1 (ref)	1.09 (1.05–1.13)
Adjusted HR^b^	1 (ref)	1.01 (0.98–1.05)
Adjusted HR including healthcare visits^c^	1 (ref)	0.99 (0.96–1.03)

CI, confidence interval; HR, hazard ratio; py, person-years.

a Stratified on matched pairs.

b Adjusted for age, sex, calendar year, county of residence, country of birth (Nordic country or other), educational level (compulsory school, upper secondary school, or college/university), ≥1 metabolic disease (diabetes, obesity, hypertension, and dyslipidaemia), chronic kidney disease, chronic obstructive pulmonary disease with diagnosis ≥40 years of age, and alcohol related disease.

c Additionally adjusted for number of healthcare visits between 6 and 24 months before start of follow-up.

### Full-sibling comparison

To address potential confounding arising from early-life environmental exposures and shared genetic factors, we matched 22 666 individuals with DD with 41 104 full-siblings without a diagnosis of DD or CVD at the index date. Individuals with DD showed a significantly increased risk of any individual MACE outcome compared with their full siblings (aHR = 1.28, 95%CI = 1.22–1.34) (*Tables S4* and *S5*).

### Stratified and sensitivity analyses

In stratified analyses of MACE, females had a higher relative risk than males [*P*_Interaction_ = 0.001; females (aHR = 1.27, 95%CI = 1.23–1.30) vs. males (1.21, 95%CI = 1.17–1.25)]. Individuals with colorectal histological inflammation showed a higher risk of MACE compared to those without inflammation [*P*_Interaction_ = 0.016; histological inflammation (aHR = 1.28, 95%CI = 1.23–1.33) vs. no inflammation (1.22, 95%CI = 1.19–1.24)]. Comparing age groups (categories: <30 years of age and thereafter subdivided in 10-year age strata), the relative risk was highest in those diagnosed with DD at young age (<30 years: aHR = 2.99, 95%CI = 1.95–4.59), and the risk estimates decreased with older age at diagnosis (*P*_Trend_ < 0.001). The risk was highest during the first year after DD diagnosis (aHR = 1.50, 95%CI = 1.40–1.60) but remained elevated thereafter [1–5 years (aHR = 1.16, 95%CI = 1.11–1.20) and >5 years (aHR = 1.25, 95%CI = 1.22–1.29) of follow-up]. (Supplementary *Table S6*)

When restricting the definition of DD to inpatient diagnosis, the aHR was 1.22 (95%CI = 1.20–1.25). The corresponding aHR for having a main diagnosis of MACE was 1.20 (95%CI = 1.18–1.23). (Supplementary material online, *Table S7*)

## Discussion

In this nationwide Swedish cohort of individuals with DD (1987–2017), we found a 24% increased relative risk of incident MACE compared with matched population reference individuals. This is equivalent to one extra MACE for every six individuals with DD followed for 10 years. The risk was highest in younger adults and females. The results were consistent after adjustment for healthcare utilization and in full-sibling comparisons.

To the best of our knowledge, this is the first study of DD and specific MACE components within the same large population-based cohort. A population-based American study by Tam *et al*. of 43 904 individuals (1986–2012) reported an elevated relative risk (aHR = 1.35, 95%CI = 1.07–1.70) of CVD (defined as fatal or non-fatal myocardial infarction and stroke) in those with a history of diverticulitis and after adjustment for several confounders, including medication.^10^ The relative risk is comparable to ours, although, their study population did not represent an average nationwide sample and was limited to only males aged 40 to 75 years. In contrast, in our nationwide sample we included individuals of any sex and aged ≥18 years with diverticulosis or diverticulitis. The risk estimates therefore represent different study populations and a full spectrum of DD events, including both diverticulosis and diverticulitis. Another difference between our studies is that our data was exclusively physician-reported, while Tam et al. also included self-reported data, potentially affected by recall bias.

Consistent with our findings, a Danish register-based matched cohort study of 77 065 incident cases of DD (1980–2011) based on ICD codes reported increased adjusted IR ratios for acute myocardial infarction (1.11, 95%CI = 1.07–1.14) and stroke (1.11, 95%CI = 1.08–1.15).^11^

The risk of acute coronary syndrome (a broad definition was used) was investigated in an Asian population of individuals with DD (*N* = 52 618, 2000–2011) and matched reference individuals, reporting an aHR of 1.23 (95%CI = 1.14–1.32).^22^ Interestingly, this study found a significantly higher risk in individuals with diverticulitis compared with diverticulosis (aHR 1.25, 95%CI = 1.15–1.37 vs. 1.19, 95%CI = 1.07–1.32; *P* < 0.001). Although we could not discriminate between diverticulitis and diverticulosis in our study, we found a slightly higher risk in individuals with a histopathology report of colonic inflammation vs. no inflammation (aHR 1.28 vs. 1.22; *P* = 0.016).

As opposed to the study by Tam *et al.*, our study also included females with DD (*n* = 32 030). The proportion of females in our study (61%) is identical to that reported in the Danish DD study on myocardial infarction and stroke ten years ago.^11^ Females had a slightly higher relative risk (aHR 1.27 vs. 1.21, *P*_Interaction_ = 0.001) compared with men. The mean age at DD diagnosis was 61 years in our study with some 81% aged ≥50 years. This is also similar to the Danish study (84% ≥50 years). Notably, we report the highest relative risk in those diagnosed at age <30 years (aHR = 2.99), with decreasing risk estimates as age at DD diagnosis increases. The risk of MACE specifically attributable to DD seems more pronounced in younger ages where comorbidities are less common. The prevalence of comorbidities associated with CVD outcomes is highest in the elderly, which may explain this trend of decreasing risks with older age at DD diagnosis.

We report increased risk of all individual MACE components, except for cardiovascular mortality. We defined cardiovascular mortality as a CVD diagnosis registered as cause of death in the CDR. The coverage of the CDR was nearly 100% during the study period, with high validity of recorded diagnoses. In a previous study,^23^ we reported a 27% (aHR: 1.27, 95%CI: 1.25–1.29) increased risk of all-cause mortality in patients with DD compared with reference individuals. The cause-specific cardiovascular mortality was 12% (aHR: 1.12, 95%CI: 1.10–1.15) after adjustment for several comorbidities (compared with HR 1.18, 95%CI: 1.16–1.21 before adjustment for cancer). Similar to our current study, the study population was identified from the ESPRESSO study applying identical diagnostic criteria for DD; however, there are differences between the studies. Since the outcome in the current study was MACE and its components, we excluded patients with DD (43%) and reference individuals (29%) with prevalent CVD (using broad exclusion criteria) before the index date. The previous study was designed to investigate all-cause mortality in DD and therefore did not exclude individuals with prior CVD; hence, the exposure to CVD was longer in that study. We believe the difference between the two studies in exclusion criteria, with shorter exposure to CVD in the current study, could explain our non-significant estimate of cardiovascular mortality.

The role of systemic inflammation in the atherosclerotic process and development of CVD in different diseases, including in GI disease, has been reported in both randomized clinical trials^24^ and epidemiological population-based studies.^8,9,25^ Chronic low-grade inflammation has been proposed to be involved in the pathogenesis of DD.^12,26^ Moreover, elevated levels of circulating C-reactive protein and interleukin 6, as markers of chronic inflammation, have been described in DD.^27,28^ We speculate that these mechanisms may harbour an increased risk of CVD outcomes in individuals with DD. Alterations in the gut microbiota and intestinal flora metabolites have been associated with chronic inflammation and negative CVD outcomes^29,30^ where current evidence supports a link between coronary artery disease^31^ and heart failure^32,33^ and the gut microbiota composition. Some studies indicate that individuals with DD have an altered microbiota compared to those without DD.^34–36^ Depletion of some gut microbiota species with anti-inflammatory properties associated with mucosal macrophage infiltration found in patients with DD can be one possible link between the microbiota and the development of DD.^34^ However, the exact role of the host microbiota in DD has not been fully elucidated, and a meta-analysis investigating current evidence on the topic concluded that there is no convincing evidence to suggest that the microbiota contributes to the pathogenesis of DD.^37^ Whether the risk of MACE shown in our study is associated with inflammatory properties of DD and an altered microbiota, and if so, to what extent, is not possible to disentangle with our study design. Nevertheless, the role of the microbiota in inducing a state of low-grade systemic inflammation potentially leading to the development of both DD and CVD could partly explain the increased risk of MACE seen in our study.

Notably, metabolic comorbidities were twice as common in patients with DD compared with the reference individuals. It is possible that the association between DD and MACE in our study is also driven by confounding attributable to CVD risk factors we did not fully account for in our statistical models. We acknowledge that our findings could indeed partly be due to residual confounding caused by a lack of data on important risk factors for DD and CVD, such as dietary habits, lipid profiles, body mass index, physical activity, medication, and granular data on smoking and alcohol use. While lack of data on these potential confounders is a weakness, when Tam et al. adjusted for eight potential confounders (red meat intake, fibre intake, body mass index, calorie intake, smoking, Alternate Healthy Eating Index, alcohol consumption, and physical activity), the HR for incident CVD decreased by less than 10% (from 1.49 to 1.40), suggesting these factors have a limited impact on the association between DD and CVD. Consistent with previous findings,^10^ we report statistically significant increased risk also after adjustment for diabetes, dyslipidaemia, obesity, hypertension, COPD (as a proxy for heavy smoking, also used in the Danish study by Strate *et al.*^11^), and alcohol-related diseases. Moreover, the association between DD and MACE remained statistically significant after addressing surveillance bias, where we adjusted for the number of healthcare visits 6–24 months before baseline. Similarly, in the American study, the results were similar also in a subgroup of patients with regular physical examinations.^10^ Our results were further corroborated in full-sibling comparison, partly accounting for genetic and early life environmental exposure, and remained largely unchanged in a sensitivity analysis using stricter definitions of DD (requiring DD as primary diagnosis or inpatient diagnosis). Future research should focus on examining underlying pathophysiological mechanisms and potential shared risk factors between DD and CVD. Among these, hypertension has been identified as a risk factor for hospital visits for DD.^38^

Our study has several strengths. It encompasses a study population of individuals diagnosed with DD sourced from nationwide registers, which offer nearly full coverage and with virtually no loss to follow-up. These registers have previously demonstrated high validity of the included covariates. Moreover, the DD diagnosis has been validated in a Swedish setting with a positive predictive value of >90%.^39^ Through our case ascertainment with colorectal histopathology reports in the ESPRESSO study, we assume the validity of our case definition to be potentially even higher. The use of histopathology data enabled us to distinguish those with a history of histological inflammation in the colon from those without; however, we were not able to discriminate between generic colonic histological inflammation and inflammation in diverticula. Moreover, diagnosis of individual MACE components shows high validity (PPVs >90%) in the registers, including heart failure,^40^ acute coronary syndromes,^41,42^ and stroke.^43^ The Swedish healthcare system is tax-funded and provides equal access to healthcare to all legal residents. Leveraging a nationwide register with near-complete coverage of the total Swedish population and with a study period of over 30 years, we expect limited selection bias. The prospective collection of data used in our study also minimized recall bias, and the large sample size allowed us to assess the risk associated with MACE through various sensitivity analyses, including full-sibling comparison, to reduce the influence of shared genetics and early-life environmental factors.

We acknowledge some limitations to our study. First, our case definition of DD included colorectal histopathology, thus, not capturing DD in individuals without endoscopic examination with a biopsy. However, the potential influence of this restriction is likely small since guidelines have traditionally recommended follow-up with colonoscopy after DD diagnosis.^44^ Moreover, a recent large population-based Danish study (with a similar healthcare system as Sweden) demonstrated that over two-thirds of patients with DD undergo colonoscopy prior to diagnosis and another 15% surgery for DD (our study also included histopathology from surgical specimens).^45^ Second, we cannot exclude the existence of false-negative DD in the reference population. However, such potential misclassification would likely have attenuated our association. Third, we could not distinguish the severity of DD between the exposed (uncomplicated vs. complicated or asymptomatic vs. symptomatic). It is possible that those with an indication of a colonoscopy had a more severe DD and that mild cases of DD could have been misclassified as non-DD. This could have biased our results to underestimate the true risk of MACE in patients with DD. Fourth, although we adjusted for several important covariates, the reported association may partly be due to residual confounding due to lifestyle factors. As already described, we lacked granular data on some known risk factors for DD and CVD. Finally, the study population was of predominantly Caucasian origins, and our results may not be generalizable to other ethnic groups.

In summary, our study revealed a 24% higher relative risk of MACE in patients with DD than in reference individuals. This is equivalent to one extra case of MACE in every six patients with DD followed for 10 years. These excess risks persisted after accounting for comorbidities, healthcare utilization, and when comparing with DD-free siblings. Future research should confirm our findings and examine underlying pathophysiological mechanisms contributing to the elevated CVD risk, including potentially shared risk factors between DD and CVD. Considering the high underlying incidence and prevalence of cardiovascular morbidity-mortality in the general population, even the moderately elevated risk of MACE among individuals with DD shown in our study emphasizes the importance of awareness of CVD and its risk factors in this group of patients.

## Supplementary Material

qcae074_Supplemental_File

## Data Availability

The data underlying this article cannot be shared publicly due to Swedish regulations.
