# Supplementary material for: Diverticular disease and risk of incident major adverse cardiovascular events: a nationwide matched cohort study
Source: Eur Heart J Qual Care Clin Outcomes. 2024 Aug 22;11(4):415–23. doi: 10.1093/ehjqcco/qcae074 (PMC12187049; doi:10.1093/ehjqcco/qcae074)
Supplement: qcae074_Supplemental_File [file qcae074_supplemental_file.docx]

**Supplementary Material**

This supplement contains additional tables and figures to the study by Anders Forss et al:

"Diverticular disease and risk of incident major adverse cardiovascular events: A nationwide matched cohort study"

**Table of contents**

**Table S1.** Study exclusion criteria (page 2)

**Table S2.** Definitions of primary and secondary outcomes (page 3)

**Table S3.** Definitions of covariates (page 4)

**Table S4.** Cohort characteristics in individuals with diverticular disease and full-siblings at the start of follow-up (1987-2017) (pages 5-6)

**Table S5.** Stratified analyses of incidence rates and hazard ratios for incident major adverse cardiovascular events in individuals with diverticular disease compared with their full-siblings (1987-2021) (pages 7-8)

**Table S6.** Stratified analyses of incidence rates and hazard ratios for incident major adverse cardiovascular events in individuals with diverticular disease compared with general population reference individuals (1987-2021) (pages 9-10)

**Table S7.** Incidence rates and hazard ratios for incident major adverse cardiovascular events as main and inpatient diagnosis in individuals with diverticular disease compared with general population reference individuals (1987-2021) (Page 11)

**Figure S1**. Flowchart of study exclusion and inclusion (1987-2017) (page 12)

**Figure S2 A-D.** Cumulative incidence plots and 95% confidence intervals of individual incident major adverse cardiovascular events (1987-2021) (pages 13-14)

**Table S1.** Study exclusion criteria

|  | **Excluded conditions** |  | **Definition^a^** |  | **Criteria** |
| --- | --- | --- | --- | --- | --- |
|  | **Inflammatory bowel diseases** | ICD-7/8 | ICD-9 | ICD-10 | Exclude if ≥2 ICD codes |
|  | Ulcerative colitis |  | 556 | K51 |  |
|  | Crohn’s disease |  | 555 | K50 |  |
|  | IBD unclassified |  |  | K52.3 |  |
|  | **Cardiovascular disease** | ICD-7/8 | ICD-9 | ICD-10 | Exclude if ≥1 ICD code |
|  |  | 394-399; 410-445; Procedure code op-6; 3065, 3066, 3105, 3127, 3158, 3092, 3080, 3068, 3085, 3149 | 391-400; 406-459; Procedure code op-6; 3065, 3066, 3105, 3127, 3158, 3092, 3080, 3068, 3085, 3149 | I00-I99 (not I10 and 11.9-I16); G45; Procedure code op-6, op-7;  F procedure codes |  |
|  | **Previous colectomy** |  | Procedure code (KVÅ) | Procedure code (NOMESCO) | Exclude if ≥1 procedure code |
|  |  |  | 4650, 4651, 4652, 4653, 4654, 4793, 4794, 4795, 4822, 4823 | JFC40, JFC41, JFG53, JFG60, JFG70, JFG73, JFG80, JFG86, JFG96, JFH00, JFH01, JFH10, JFH11, JFH20-JFH40, JFH96, JGB40, JGB50, JGB60, JGB61 |  |

**ICD, international classification of disease; KVÅ, (in Swedish, klassifikation av vårdåtgärder), a Swedish classification of surgical and medical procedures up until 1997; NOMESCO, nomenclature of medicine, an adapted version of the NOMESCO Classification of Surgical Procedures used from 1997 onwards.**

^a^ **Hospital inpatient discharge letters or outpatient specialty care notes in the Swedish National Patient Register. If recorded in the registers on or before the index biopsy date for microscopic colitis**

**Table S2** Definitions of primary and secondary outcomes

|  | **Outcome^a^** | **Definition** | **Data source and criteria** |
| --- | --- | --- | --- |
|  | **Primary** |  |  |
|  | Major adverse cardiovascular event | Composite outcome including any of the following secondary outcomes (defined below):  (1) ischemic heart disease (including acute myocardial infarction)  (2) congestive heart failure  (3) stroke  (4) cardiovascular death |  |
|  | **Secondary** |  | Hospital inpatient discharge letters or outpatient specialty care notes in the Swedish National Patient Register. |
|  | Ischemic heart disease |  | ICD-8/9: 410-414; ICD-10: I20-25 |
|  | Congestive heart failure |  | ICD-8: 427,00, 427,10; ICD-9: 428A, 428B, 428X; ICD-10: I11.0, I50 |
|  | Stroke |  | ICD-8/9: 430-438; ICD-10: I60-64 |
|  | Cardiovascular mortality | Cause of Death Register (primary cause of death) | ICD-8: 390-458; ICD-9: 390-459; ICD-10: I00-I99 |

**ICD, international classification of disease**

**^a^ If recorded in the registers after index date of diverticular disease.**

**Table S3** Definitions of covariates

|  | **Comorbidities^a^** | **ICD-7/8** | **ICD-9** | **ICD-10** |  |
| --- | --- | --- | --- | --- | --- |
|  | Diabetes | 250 | 250 | E10.0-E14.9 |  |
|  | Obesity | 278, 649,1, 649B | 278, 649,1, 649B | E65-66 |  |
|  | Hypertension | - | 400-404; 401-405 | I10-I16 (not I11.0) |  |
|  | Dyslipidemia | 272 | 272 | E78 |  |
|  | Chronic kidney disease | 585, 586, Y29,01 | 585, 586, 753B, V42A, V45B, V56 | N18-23; N26; T82.4; Y84.1; Q61; Z49; Z99.2; Z94.0 |  |
|  | Chronic obstructive pulmonary disease | 491, 492, 496 | 491, 492, 496 | J41-44 |  |
|  | Alcohol related disease |  |  |  | Definitions used presented elsewhere^b^ |
|  | | | | | |

ICD, international classification of disease; SNOMED, systematized nomenclature of medicine

**^a^ Hospital inpatient discharge letters or outpatient specialty care notes in the National Swedish Patient Register. If recorded in the registers on or before index date of diverticular disease.**

**^b^ Definitions (ICD codes) used for identifying alcohol related disease are described in detail by Bergman et al, Incidence of ICD-Based Diagnoses of**

**Alcohol-Related Disorders and Diseases from Swedish Nationwide Registers and Suggestions for Coding. Clinical Epidemiology 2020;12:1433–1442.** https://www.dovepress.com/incidence-of-icd-based-diagnoses-of-alcohol-related-disorders-and-dise-peer-reviewed-fulltext-article-CLEP**.**

**Table S4**. Cohort characteristics in individuals with diverticular disease and full-siblings at the start of follow-up (1987-2017)

|  | **Diverticular disease N=52 468** | **Full-siblings N=41 104** |
| --- | --- | --- |
| **Sex, n (%)** |  |  |
| Male | 20 438 (39) | 20 281 (49) |
| Female | 32 030 (61) | 20 823 (51) |
| **Age, years** |  |  |
| Mean (SD) | 61 (13) | 54(12) |
| Median (IQR) | 62 (5-70) | 54 (46-62) |
| Range, min-max | 18-98 | 18-83 |
| **Age groups, years, n (%)** |  |  |
| <30 | 736 (1) | 1 163 (3) |
| 30-39 | 2 571 (5) | 3 754 (9) |
| 40-49 | 6 867 (13) | 9 483 (23) |
| 50-59 | 12 452 (24) | 13 352 (32) |
| 60-69 | 15 507 (30) | 10 363 (25) |
| 70-79 | 10 706 (20) | 2 899 (7) |
| ≥ 80 | 3 629 (7) | 90 (0) |
| **Country of birth, n (%)** |  |  |
| Nordic | 49 557 (94) | 40 778 (99) |
| Other | 2 910 (6) | 325 (1) |
| NA | 1 (0) | 1 (0) |
| **Level of education, n (%)** |  |  |
| Compulsory school (≤9 years) | 16 136 (31) | 10 896 (27) |
| Upper secondary school (10-12 years) | 20 782 (40) | 18 422 (45) |
| College or university (≥13 years) | 11 246 (21) | 10 020 (24) |
| NA | 4 304 (8) | 1 766 (4) |
| **Calendar year start of follow-up** |  |  |
| 1987-1999 | 10 268 (20) | 5 733 (14) |
| 2000-2009 | 20 733 (40) | 16 618 (40) |
| 2010-2017 | 21 467 (41) | 18 753 (46) |
| **Time between first diagnosis and biopsy, years** |  |  |
| Median (IQR) | 0.4 (0.0-4.7) |  |
| **Number of healthcare visits**^a^**, n (%)** |  |  |
| 0 | 19 594 (37) | 24 290 (59) |
| 1 | 9 445 (18) | 6 371 (15) |
| 2-3 | 10 187 (19) | 5 458 (13) |
| ≥4 | 13 242 (25) | 4 985 (12) |
| **Follow-up time, years** |  |  |
| Mean (SD) | 9.7 (6.3) | 11.4 (6.4) |
| Median (IQR) | 8.6 (5.3-13.2) | 10.1 (6.7-15.2) |
| Range, min-max | 0.0-34.5 | 0.0-33.9 |
| **Follow-up time, n (%)** |  |  |
| <2 years | 4689 (9) | 1 228 (3) |
| 2 to <10 years | 26 344 (50) | 19 036 (46) |
| ≥10 years | 21 435 (41) | 20 840 (51) |
| **Comorbidity at start of follow-up, n (%)** |  |  |
| ≥1 metabolic disease^b^ | 10 581 (20.2) | 3 816 (9.3) |
| Diabetes | 2 818 (5.4) | 1 289 (3.1) |
| Obesity | 1 120 (2.1) | 586 (1.4) |
| Hypertension | 8 331 (15.9) | 2 518 (6.1) |
| Dyslipidemia | 1 378 (2.6) | 552 (1.3) |
| Chronic kidney disease | 1 883 (3.6) | 819 (2.0) |
| COPD (diagnosis at age ≥40 years) | 1 582 (3.0) | 429 (1.0) |
| Alcohol related disease | 1 739 (3.3) | 1 242 (3.0) |

COPD, chronic obstructive pulmonary disease; IQR, interquartile range; NA, data not available; SD, standard deviation

^a^ Between 6 and 24 months before start of follow-up.

^b^ Includes ≥1 of diabetes, obesity, hypertension, and dyslipidemia.

**Table S5.** Stratified analyses of incidence rates and hazard ratios for incident major adverse cardiovascular events in individuals with diverticular disease compared with their full-siblings (1987-2021)

|  | **Diverticular disease** | **Full-siblings** | **Events Diverticular disease** | **Events**  **Full-siblings** | **IR**  **Diverticular disease** | **IR**  **Full-siblings** | **Unadjusted Hazard ratio (95% CI)^a^** | **Adjusted**  **Hazard ratio**  **(95% CI)^b^** |
| --- | --- | --- | --- | --- | --- | --- | --- | --- |
| **Overall** | 22 666 (100.0) | 41 104 (100.0) | 4 625 (20.4) | 6 713 (16.3) | 19.1 (18.5-19.7) | 14.3 (13.9-14.6) | 1.34 (1.28-1.41) | 1.28 (1.22-1.34) |
| **Follow-up, years** |  |  |  |  |  |  |  |  |
| <1 | 22 666 (100.0) | 41 104 (100.0) | 334 (1.5) | 351 (0.9) | 15.0 (13.4-16.6) | 8.6 (7.7-9.5) | 1.73 (1.47-2.05) | 1.58 (1.33-1.88) |
| 1-5 | 22 073 (97.4) | 40 511 (98.6) | 1 212 (5.5) | 1 713 (4.2) | 14.4 (13.6-15.2) | 11.0 (10.5-11.5) | 1.31 (1.21-1.43) | 1.23 (1.13-1.34) |
| >5 | 19 293 (85.1) | 36 034 (87.7) | 3 079 (16.0) | 4 649 (12.9) | 22.7 (21.9-23.5) | 17.0 (16.5-17.5) | 1.32 (1.25-1.40) | 1.26 (1.19-1.34) |
| **Sex^c^** |  |  |  |  |  |  |  |  |
| Female | 12 817 (56.5) | 20 823 (50.7) | 2 262 (17.6) | 2 633 (12.6) | 16.4 (15.7-17.1) | 10.8 (10.4-11.2) | 1.42 (1.30-1.54) | 1.34 (1.24-1.46) |
| Male | 9 849 (43.5) | 20 281 (49.3) | 2 363 (24.0) | 4 080 (20.1) | 22.7 (21.8-23.7) | 18.0 (17.4-18.6) | 1.28 (1.18-1.39) | 1.22 (1.12-1.33) |
| **Age groups, years^d^** |  |  |  |  |  |  |  |  |
| <30 | 487 (2.1) | 1 163 (2.8) | 29 (6.0) | 47 (4.0) | 5.0 (3.3-7.1) | 3.0 (2.2-4.0) | 2.17 (1.06-4.44) | 2.16 (0.91-5.10) |
| 30-39 | 1 757 (7.8) | 3 754 (9.1) | 172 (9.8) | 285 (7.6) | 7.6 (6.5-8.8) | 5.4 (4.8-6.1) | 1.67 (1.23-2.26) | 1.62 (1.18-2.21) |
| 40-49 | 4 486 (19.8) | 9 483 (23.1) | 699 (15.6) | 1 150 (12.1) | 12.2 (11.3-13.1) | 9.0 (8.5-9.6) | 1.49 (1.29-1.73) | 1.44 (1.24-1.67) |
| 50-59 | 7 285 (32.1) | 13 352 (32.5) | 1 571 (21.6) | 2 333 (17.5) | 18.9 (18.0-19.9) | 14.9 (14.3-15.5) | 1.37 (1.24-1.52) | 1.31 (1.18-1.45) |
| 60-69 | 6 617 (29.2) | 10 363 (25.2) | 1 636 (24.7) | 2 163 (20.9) | 27.4 (26.1-28.8) | 22.3 (21.4-23.3) | 1.33 (1.21-1.47) | 1.25 (1.13-1.38) |
| 70-79 | 1 976 (8.7) | 2 899 (7.1) | 504 (25.5) | 717 (24.7) | 37.3 (34.1-40.7) | 34.8 (32.3-37.5) | 1.13 (0.92-1.38) | 1.08 (0.87-1.33) |
| ≥80 | 58 (0.3) | 90 (0.2) | 14 (24.1) | 18 (20.0) | 57.9 (31.6-97.1) | 39.4 (23.4-62.3) | - (0.00-Inf) | - (0.00-Inf) |
| **Calendar year start of follow-up** |  |  |  |  |  |  |  |  |
| 1987-1999 | 2 721 (12.0) | 5 733 (13.9) | 1 042 (38.3) | 1 619 (28.2) | 19.8 (18.6-21.0) | 13.8 (13.1-14.4) | 1.34 (1.24-1.45) | 1.29 (1.19-1.40) |
| 2000-2009 | 8 982 (39.6) | 16 618 (40.4) | 2 189 (24.4) | 3 212 (19.3) | 19.2 (18.5-20.1) | 14.6 (14.1-15.1) | 1.30 (1.23-1.37) | 1.25 (1.18-1.32) |
| 2010-2017 | 10 963 (48.4) | 18 753 (45.6) | 1 394 (12.7) | 1 882 (10.0) | 18.4 (17.5-19.4) | 14.2 (13.6-14.9) | 1.29 (1.20-1.38) | 1.20 (1.12-1.29) |
| **Country of birth** |  |  |  |  |  |  |  |  |
| Nordic | 22 485 (99.2) | 40 778 (99.2) | 4 593 (20.4) | 6 676 (16.4) | 19.1 (18.6-19.7) | 14.3 (14.0-14.6) | 1.34 (1.28-1.41) | 1.28 (1.22-1.34) |
| Other | 181 (0.8) | 325 (0.8) | 32 (17.7) | 37 (11.4) | 17.6 (12.0-24.8) | 10.9 (7.7-15.0) | 1.46 (0.70-3.07) | 1.39 (0.64-3.04) |
| **Level of education, years** |  |  |  |  |  |  |  |  |
| Compulsory school (≤9) | 5 637 (24.9) | 10 896 (26.5) | 1 515 (26.9) | 2 444 (22.4) | 25.3 (24.0-26.6) | 19.6 (18.8-20.4) | 1.28 (1.20-1.37) | 1.21 (1.14-1.30) |
| Upper secondary school (10-12) | 10 340 (45.6) | 18 422 (44.8) | 1 992 (19.3) | 2 717 (14.7) | 18.2 (17.4-19.0) | 13.1 (12.6-13.6) | 1.33 (1.26-1.41) | 1.25 (1.18-1.33) |
| College or university (≥13) | 5 843 (25.8) | 10 020 (24.4) | 879 (15.0) | 1 175 (11.7) | 14.3 (13.3-15.2) | 10.6 (10.0-11.2) | 1.29 (1.18-1.41) | 1.20 (1.10-1.31) |
| NA | 846 (3.7) | 1 766 (4.3) | 239 (28.3) | 377 (21.3) | 21.5 (18.8-24.4) | 13.6 (12.3-15.1) | 1.60 (1.36-1.88) | 1.53 (1.30-1.81) |
| **Diverticular disease** |  |  |  |  |  |  |  |  |
| Diagnosis before biopsy | 15 614 (68.9) | 28 709 (69.8) | 3 250 (20.8) | 4 802 (16.7) | 18.7 (18.0-19.3) | 14.0 (13.6-14.4) | 1.29 (1.24-1.35) | 1.23 (1.18-1.29) |
| Biopsy before diagnosis | 6 591 (29.1) | 11 499 (28.0) | 1 293 (19.6) | 1 770 (15.4) | 20.2 (19.1-21.4) | 14.8 (14.1-15.5) | 1.34 (1.24-1.44) | 1.28 (1.19-1.37) |
| **Comorbidity at start of follow-up** |  |  |  |  |  |  |  |  |
| Diabetes | 996 (4.4) | 1 289 (3.1) | 314 (31.5) | 376 (29.2) | 41.5 (37.0-46.4) | 35.9 (32.4-39.7) | 1.18 (1.01-1.37) | 1.16 (1.00-1.35) |
| Dyslipidemia | 609 (2.7) | 552 (1.3) | 167 (27.4) | 119 (21.6) | 36.5 (31.2-42.5) | 29.4 (24.3-35.1) | 1.29 (1.01-1.63) | 1.25 (0.98-1.58) |
| Obesity | 580 (2.6) | 586 (1.4) | 128 (22.1) | 111 (18.9) | 26.8 (22.4-31.9) | 22.2 (18.3-26.8) | 1.34 (1.04-1.73) | 1.35 (1.04-1.74) |
| Hypertension | 3 196 (14.1) | 2 518 (6.1) | 860 (26.9) | 606 (24.1) | 33.9 (31.7-36.3) | 30.9 (28.5-33.4) | 1.15 (1.04-1.28) | 1.16 (1.04-1.28) |
| Chronic kidney disease | 843 (3.7) | 819 (2.0) | 217 (25.7) | 154 (18.8) | 33.2 (29.0-38.0) | 22.9 (19.4-26.8) | 1.47 (1.19-1.81) | 1.39 (1.13-1.72) |
| COPD (diagnosis at age ≥40 years) | 489 (2.2) | 429 (1.0) | 168 (34.4) | 113 (26.3) | 49.5 (42.3-57.6) | 35.4 (29.2-42.6) | 1.48 (1.16-1.89) | 1.44 (1.12-1.84) |
| Alcohol related disease | 838 (3.7) | 1 242 (3.0) | 232 (27.7) | 313 (25.2) | 30.7 (26.9-34.9) | 26.8 (24.0-30.0) | 1.17 (0.99-1.39) | 1.13 (0.95-1.35) |
| **Inflammation in histopathology^e^** |  |  |  |  |  |  |  |  |
| Yes | 4 443 (19.6) | 8 222 (20.0) | 992 (22.3) | 1 433 (17.4) | 20.3 (19.0-21.6) | 14.4 (13.6-15.1) | 1.38 (1.27-1.50) | 1.32 (1.22-1.44) |
| No | 18 223 (80.4) | 32 882 (80.0) | 3 633 (19.9) | 5 280 (16.1) | 18.8 (18.2-19.4) | 14.2 (13.9-14.6) | 1.29 (1.24-1.35) | 1.23 (1.18-1.28) |

Values are n (%), unless otherwise indicated.

COPD, chronic obstructive pulmonary disease; IR, incidence rate; NA, data not available

^a^ Stratified on matched pairs.

^b^ Adjusted for age, sex, calendar year, county of residence, country of birth (Nordic country or other), educational level (compulsory school, upper secondary school, or college/university), ≥1 metabolic disease (diabetes, obesity, hypertension, and dyslipidemia), chronic kidney disease, chronic obstructive pulmonary disease with diagnosis ≥40 years of age, and alcohol related disease.

**Table S6.** Stratified analyses of incidence rates and hazard ratios for incident major adverse cardiovascular events in patients with diverticular disease compared to general population reference individuals (1987-2021)

|  | **Diverticular disease** | **Reference individuals** | **Events Diverticular disease** | **Events Reference individual** | **IR**  **Diverticular disease** | **IR**  **Reference individuals** | **Unadjusted Hazard ratio (95% CI)^a^** | **Adjusted**  **Hazard ratio**  **(95% CI)^b^** |
| --- | --- | --- | --- | --- | --- | --- | --- | --- |
| **Overall** | 52 468 (100.0) | 194 525 (100.0) | 16 147 (30.8) | 48 134 (24.7) | 31.7 (31.2-32.1) | 22.4 (22.2-22.6) | 1.33 (1.30-1.36) | 1.24 (1.22-1.27) |
| **Follow-up, years** |  |  |  |  |  |  |  |  |
| <1 | 52 468 (100.0) | 194 525 (100.0) | 1 511 (2.9) | 2 980 (1.5) | 29.8 (28.3-31.3) | 15.5 (15.0-16.1) | 1.61 (1.51-1.72) | 1.50 (1.40-1.60) |
| 1-5 | 49 600 (94.5) | 189 782 (97.6) | 4 702 (9.5) | 12 749 (6.7) | 25.7 (24.9-26.4) | 17.8 (17.5-18.1) | 1.26 (1.21-1.30) | 1.16 (1.11-1.20) |
| >5 | 40 713 (77.6) | 163 075 (83.8) | 9 934 (24.4) | 32 405 (19.9) | 36.0 (35.3-36.7) | 26.1 (25.9-26.4) | 1.33 (1.30-1.37) | 1.26 (1.22-1.29) |
| **Sex^c^** |  |  |  |  |  |  |  |  |
| Female | 32 030 (61.0) | 120 278 (61.8) | 9 595 (30.0) | 28 971 (24.1) | 30.5 (29.9-31.2) | 21.7 (21.4-21.9) | 1.36 (1.33-1.40) | 1.27 (1.23-1.30) |
| Male | 20 438 (39.0) | 74 247 (38.2) | 6 552 (32.1) | 19 163 (25.8) | 33.5 (32.6-34.3) | 23.7 (23.3-24.0) | 1.28 (1.24-1.33) | 1.21 (1.17-1.25) |
| **Age groups, years^d^** |  |  |  |  |  |  |  |  |
| <30 | 736 (1.4) | 3 378 (1.7) | 43 (5.8) | 73 (2.2) | 4.9 (3.5-6.6) | 1.7 (1.4-2.2) | 3.01 (2.03-4.45) | 2.99 (1.95-4.59) |
| 30-39 | 2 571 (4.9) | 11 793 (6.1) | 251 (9.8) | 623 (5.3) | 7.8 (6.8-8.8) | 4.0 (3.7-4.4) | 1.99 (1.71-2.32) | 1.86 (1.59-2.19) |
| 40-49 | 6 867 (13.1) | 30 470 (15.7) | 1 122 (16.3) | 3 408 (11.2) | 13.1 (12.3-13.9) | 8.4 (8.1-8.7) | 1.58 (1.47-1.70) | 1.45 (1.35-1.57) |
| 50-59 | 12 452 (23.7) | 51 253 (26.3) | 2 956 (23.7) | 9 236 (18.0) | 21.0 (20.3-21.8) | 14.7 (14.4-15.0) | 1.45 (1.39-1.52) | 1.34 (1.28-1.40) |
| 60-69 | 15 507 (29.6) | 56 817 (29.2) | 5 065 (32.7) | 16 027 (28.2) | 34.8 (33.9-35.8) | 27.2 (26.7-27.6) | 1.30 (1.26-1.35) | 1.22 (1.17-1.26) |
| 70-79 | 10 706 (20.4) | 32 285 (16.6) | 4 786 (44.7) | 14 176 (43.9) | 60.7 (59.0-62.4) | 51.5 (50.7-52.4) | 1.23 (1.18-1.28) | 1.16 (1.12-1.21) |
| ≥80 | 3 629 (6.9) | 8 529 (4.4) | 1 924 (53.0) | 4 591 (53.8) | 104.9 (100.3-109.7) | 92.0 (89.3-94.7) | 1.14 (1.07-1.23) | 1.08 (1.01-1.16) |
| **Calendar year start of follow-up** |  |  |  |  |  |  |  |  |
| 1987-1999 | 10 268 (19.6) | 41 976 (21.6) | 5 539 (53.9) | 19 652 (46.8) | 39.7 (38.7-40.8) | 29.1 (28.7-29.6) | 1.31 (1.27-1.35) | 1.26 (1.22-1.30) |
| 2000-2009 | 20 733 (39.5) | 77 584 (39.9) | 7 119 (34.3) | 20 124 (25.9) | 31.3 (30.5-32.0) | 21.2 (20.9-21.5) | 1.33 (1.30-1.37) | 1.24 (1.20-1.27) |
| 2010-2017 | 21 467 (40.9) | 74 965 (38.5) | 3 489 (16.3) | 8 358 (11.1) | 24.4 (23.6-25.2) | 16.0 (15.6-16.3) | 1.33 (1.28-1.38) | 1.19 (1.14-1.24) |
| **Country of birth** |  |  |  |  |  |  |  |  |
| Nordic | 49 557 (94.5) | 176 944 (91.0) | 15 484 (31.2) | 45 021 (25.4) | 32.1 (31.6-32.6) | 22.9 (22.6-23.1) | 1.34 (1.31-1.37) | 1.25 (1.22-1.27) |
| Other | 2 910 (5.5) | 17 575 (9.0) | 663 (22.8) | 3 112 (17.7) | 24.0 (22.2-25.9) | 17.6 (16.9-18.2) | 1.34 (1.06-1.68) | 1.28 (1.01-1.63) |
| **Level of education, years** |  |  |  |  |  |  |  |  |
| Compulsory school (≤9) | 16 136 (30.8) | 56 509 (29.0) | 6 483 (40.2) | 19 805 (35.0) | 42.7 (41.6-43.7) | 32.6 (32.1-33.0) | 1.25 (1.21-1.28) | 1.17 (1.14-1.21) |
| Upper secondary school (10-12) | 20 782 (39.6) | 74 783 (38.4) | 5 477 (26.4) | 15 001 (20.1) | 26.4 (25.7-27.1) | 18.2 (18.0-18.5) | 1.36 (1.32-1.40) | 1.26 (1.22-1.30) |
| College or university (≥13) | 11 246 (21.4) | 48 241 (24.8) | 2 237 (19.9) | 6 794 (14.1) | 19.8 (19.0-20.7) | 12.7 (12.4-13.0) | 1.34 (1.28-1.41) | 1.24 (1.19-1.31) |
| NA | 4 304 (8.2) | 14 992 (7.7) | 1 950 (45.3) | 6 534 (43.6) | 51.1 (48.9-53.5) | 35.6 (34.7-36.4) | 1.39 (1.32-1.47) | 1.30 (1.24-1.37) |
| **Diverticular disease** |  |  |  |  |  |  |  |  |
| Diagnosis before biopsy | 35 935 (68.5) | 138 051 (71.0) | 11 356 (31.6) | 35 445 (25.7) | 31.2 (30.6-31.8) | 22.4 (22.2-22.7) | 1.28 (1.25-1.30) | 1.21 (1.18-1.23) |
| Biopsy before diagnosis | 15 702 (29.9) | 53 183 (27.3) | 4 610 (29.4) | 12 109 (22.8) | 33.2 (32.2-34.1) | 22.6 (22.2-23.0) | 1.35 (1.31-1.40) | 1.27 (1.22-1.31) |
| **Comorbidity at start of follow-up** |  |  |  |  |  |  |  |  |
| Diabetes | 2 818 (5.4) | 6 083 (3.1) | 1 143 (40.6) | 2 224 (36.6) | 59.4 (56.0-63.0) | 47.2 (45.3-49.2) | 1.17 (1.09-1.25) | 1.16 (1.08-1.25) |
| Dyslipidemia | 1 378 (2.6) | 2 202 (1.1) | 435 (31.6) | 513 (23.3) | 43.7 (39.7-48.1) | 31.6 (29.0-34.5) | 1.34 (1.18-1.52) | 1.30 (1.14-1.48) |
| Obesity | 1 120 (2.1) | 1 744 (0.9) | 280 (25.0) | 345 (19.8) | 31.3 (27.8-35.2) | 24.0 (21.5-26.6) | 1.35 (1.15-1.58) | 1.30 (1.11-1.53) |
| Hypertension | 8 331 (15.9) | 11 845 (6.1) | 3 003 (36.0) | 3 743 (31.6) | 49.7 (47.9-51.5) | 42.0 (40.7-43.4) | 1.15 (1.10-1.21) | 1.14 (1.09-1.20) |
| Chronic kidney disease | 1 883 (3.6) | 2 941 (1.5) | 586 (31.1) | 677 (23.) | 42.9 (39.5-46.5) | 29.0 (26.9-31.3) | 1.33 (1.19-1.49) | 1.23 (1.10-1.38) |
| COPD (diagnosis at age ≥40 years) | 1 582 (3.0) | 2 038 (1.0) | 687 (43.4) | 785 (38.5) | 71.5 (66.3-77.1) | 57.6 (53.7-61.8) | 1.21 (1.09-1.34) | 1.20 (1.08-1.33) |
| Alcohol related disease | 1 739 (3.3) | 4 086 (2.1) | 588 (33.8) | 1 210 (29.6) | 41.7 (38.4-45.2) | 33.2 (31.3-35.1) | 1.27 (1.15-1.40) | 1.18 (1.07-1.31) |
| **Inflammation in histopathology^e^** |  |  |  |  |  |  |  |  |
| Yes | 11 130 (21.2) | 40 847 (21.0) | 3 867 (34.7) | 11 514 (28.2) | 36.1 (34.9-37.2) | 24.8 (24.4-25.3) | 1.36 (1.31-1.41) | 1.28 (1.23-1.33) |
| No | 41 338 (78.8) | 153 678 (79.0) | 12 280 (29.7) | 36 620 (23.8) | 30.5 (29.9-31.0) | 21.7 (21.5-22.0) | 1.29 (1.27-1.32) | 1.22 (1.19-1.24) |

Values are n (%), unless otherwise indicated.

COPD, chronic obstructive pulmonary disease; IR, incidence rate; NA, data not available

^a^ Stratified on matched pairs.

^b^ Adjusted for age, sex, calendar year, county of residence, country of birth (Nordic country or other), educational level (compulsory school, upper secondary school, or college/university), ≥1 metabolic disease (diabetes, obesity, hypertension, and dyslipidemia), chronic kidney disease, chronic obstructive pulmonary disease with diagnosis ≥40 years of age, and alcohol related disease.

^c^ P-value for interaction = 0.001.

^d^ P-value for trend across age groups <0.001.

^e^ P-value for interaction = 0.016.

**Table S7**. Incidence rates and hazard ratios for incident major adverse cardiovascular events as main and inpatient diagnosis in patients with diverticular disease compared with general population reference individuals (1987-2021)

|  | **Reference individuals**  **N=194 525** | **Diverticular disease**  **N=52 468** |
| --- | --- | --- |
| **MACE^a^** |  |  |
| **Main diagnosis** |  |  |
| Incident events, n (%) | 42 467 (21.8) | 13 901 (26.5) |
| Incidence rate/1000 py (95% CI) | 38.2 (37.9-38.6) | 51.8 (51.0-52.7) |
| Absolute rate difference/1000 py (95% CI) | 0 (ref) | 13.6 (13.1-14.1) |
| Unadjusted HR | 1 (ref) | 1.40 (1.38-1.43) |
| Stratified HR^b^ | 1 (ref) | 1.28 (1.25-1.31) |
| Adjusted HR^c^ | 1 (ref) | 1.20 (1.18-1.23) |
| Adjusted HR^d^ including healthcare visits | 1 (ref) | 1.17 (1.14-1.19) |
| **Inpatient diagnosis** |  |  |
| Incident events, n (%) | 45 151 (23.2) | 15 085 (28.8) |
| Incidence rate/1000 py (95% CI) | 40.8 (40.4-41.2) | 56.6 (55.7-57.5) |
| Absolute rate difference/1000 py (95% CI) | 0 (ref) | 15.8 (15.3-16.3) |
| Unadjusted HR | 1 (ref) | 1.44 (1.41-1.46) |
| Stratified HR^b^ | 1 (ref) | 1.31 (1.28-1.34) |
| Adjusted HR^c^ | 1 (ref) | 1.22 (1.20-1.25) |
| Adjusted HR^d^ including healthcare visits | 1 (ref) | 1.18 (1.16-1.21) |
|  |  |  |

CI, confidence interval; HR, hazard ratio; MACE, major adverse cardiovascular events; py, person-years

^a^ Includes ischemic heart disease, congestive heart failure, stroke, and cardiovascular mortality.

^b^ Stratified on matched pairs.

^c^ Adjusted for age, sex, calendar year, county of residence, country of birth (Nordic country or other), educational level (compulsory school, upper secondary school, or college/university), ⩾1 metabolic disease (diabetes, obesity, hypertension, or dyslipidemia), chronic kidney disease, chronic obstructive pulmonary disease with diagnosis ≥40 years of age, and alcohol related disease.

^d^ Also adjusted for number of healthcare visits between 6 and 24 months before start of follow-up.

**A) B)**


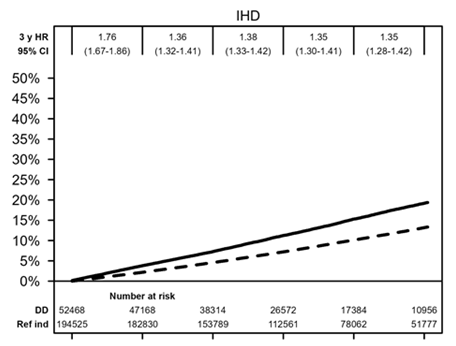

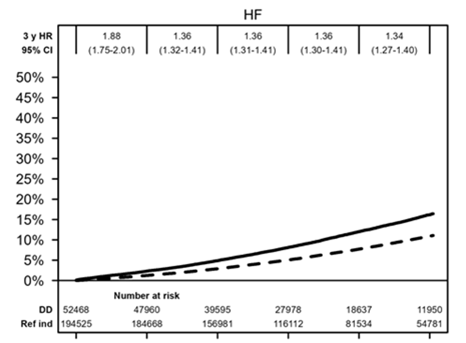


C) D)


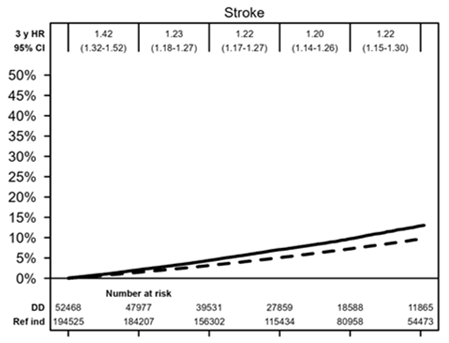

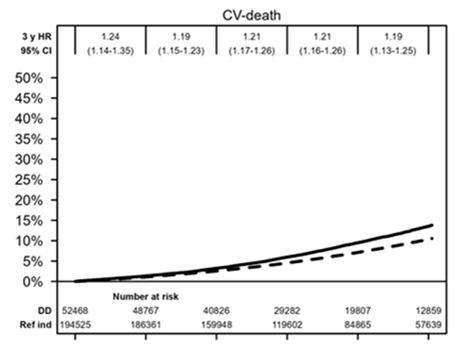


**^________^ = Diverticular disease - - - - - - - = Reference individuals**

**Figure S2 A-D**. Kaplan-Meier curves (unadjusted) of the cumulative incidence of specific major adverse cardiovascular events and hazard ratios (unadjusted) for patients with Diverticular disease compared with population reference individuals per three-year periods during 15 years of follow-up (1987 onwards). (A) IHD, ischemic heart disease; (B) HF, congestive heart failure; (C) stroke; and (D) CV-death, cardiovascular mortality. The y-axis shows the cumulative event risk and the x-axis number of follow-up years (0-15 years).

CI, confidence interval; HR, hazard ratio
